# Expression, Purification, and Evaluation of Diagnostic Potential and Immunogenicity of a Recombinant NS3 Protein from All Serotypes of Dengue Virus

**DOI:** 10.1155/2012/956875

**Published:** 2012-12-03

**Authors:** Laura Mónica Álvarez-Rodríguez, Angel Ramos-Ligonio, José Luis Rosales-Encina, María Teresa Martínez-Cázares, Aurora Parissi-Crivelli, Aracely López-Monteon

**Affiliations:** ^1^LADISER Inmunología y Biología Molecular, Facultad de Ciencias Químicas, Universidad Veracruzana, 94340 Orizaba, VER, Mexico; ^2^Centro de Investigaciones Biomédicas, Universidad Veracruzana, 91190 Xalapa, VER, Mexico; ^3^Laboratorio de Biología Molecular, Departamento de Infectómica y Patogénesis Molecular, Centro de Investigaciones y de Estudios Avanzados del Instituto Politécnico Nacional, 07360 México, DF, Mexico; ^4^Laboratorio Estatal de Salud Pública, Secretaría de Salud de Veracruz, 91697 Xalapa, VER, Mexico

## Abstract

Dengue is one of the major public health concerns in the world. Since all the four serotypes are actively circulating in Mexico, there is a need to develop an efficient diagnosis system to improve case management of the patients. There exist few studies evaluating the use of the NS3 protein as a protective antigen against dengue virus (DENV). In this paper we show the expression of a recombinant NS3 protein from all serotypes of dengue virus (GST-DVNS3-1-4) and report a reliable “in-house detection system” for the diagnosis of dengue infection which was field-tested in a small village (Tezonapa) in the state of Veracruz, Mexico. The fusion proteins were immunogenic, inducing antibodies to be able to recognize to antigens up to a 1 : 3200 dilution. The purified proteins were used to develop an in-house detection system (ELISA) and were further tested with a panel of 239 serum samples. The in-house results were in excellent agreement with the commercial kits with *κ* = 0.934 ± 0.064 (95%  CI = 0.808–1.061), and *κ* = 0.872 ± 0.048 (95%  CI = 0.779–0.965) for IgM and IgG, respectively. The agreement between the NS1 antigen detection versus the rNS3 ELISA, *κ* = 0.837 ± 0.066 (95%  CI = 0.708–0.966), was very good. Thus, these results demonstrate that recombinant NS3 proteins have potential in early diagnosis of dengue infections.

## 1. Introduction

Dengue virus (DENV) infection in America, as in the rest of the world, is increasing dramatically. Currently, Mexico could be considered as an endemic region for dengue since the mosquito vector *Aedes aegypti* is present in more than 85% of the country [1]. Infection can lead to dengue fever (DF), a self-limiting febrile illness. A more severe form of the disease is dengue hemorrhagic fever/dengue shock syndrome (DHF/DSS) with fatal consequences. Dengue consists of four, closely related but antigenically, distinct viral serotypes (DENV1–4) [2]. It is well documented that primary infection with one of the four serotypes confers long-lasting immunity to that specific serotype. However, secondary infection with a different serotype is associated with an increased risk of developing DHF where an antibody-dependent enhancement (ADE) of infection is associated with the pathophysiological mechanisms of DHF [3, 4].

The viral genome contains a single open reading frame that codes for a polyprotein of 3391 amino acids, which is processed into 10 individual proteins. Three of these proteins are structural (membrane (M), capsid (C), and envelope (E)) and 7 of them are nonstructural (NS1, NS2A, NS2B, NS3, NS4A, NS4B, and NS5) [5–7]. The cleavage of this polyprotein, which represents an essential step for viral replication, is performed by host enzymes and the NS3 viral protease. The dengue non-structural 3 (NS3) is a multifunctional protein of approximately 69 kDa, involved in the polyprotein processing, RNA capping, and RNA replication. It contains a serine-protease domain, located at the N-terminal portion, and a helicase [8]. The dengue infection elicits different immune responses towards the viral proteins. Antibodies are generated mainly against the virus surface E protein and the secreted NS1 protein [9–11], while the majority of T-cell epitopes are concentrated within the NS3 protein, the main target for CD4^+^ and CD8^+^ T-cell response [11–13]. The E protein may also induce non-neutralizing antibodies involved in the phenomenon of antibody-dependent enhancement (ADE) of DENV infection, which can be associated to the occurrence of increased numbers of DHF in secondary infections [3, 14]. Alternatively, some reports suggest the use of non-structural proteins for dengue vaccines to overcome such problem [15–17]. The NS1 is also highly immunogenic [18]; however antibodies against the NS1 may also cross-react with human proteins, which can be associated to some pathological effects of the dengue infection [19–21]. In contrast, there are only few studies evaluating the use of the NS3 protein as a protective antigen against DENV.

It is has been estimated that there are more than 3.6 billion people at risk of dengue infection with 36 million cases of dengue fever, more than 2 million cases of severe dengue, and more than 21,000 deaths occurring each year [22].

Since all the four serotypes are circulating in Mexico, there is a need to develop an efficient diagnosis system to improve case management of the patients. Until now, the incidence of dengue infection has been underestimated since most cases are not properly diagnosed, especially in small towns or villages where private or state laboratories for diagnosis are lacking [23]. According to this, early diagnosis during acute infection is critical to clinically manage severe disease and to identify potential outbreaks in a timely manner. Dengue infection diagnosis can be achieved by several assays such as RT-PCR [24], virus isolation [25], and NS1 antigen detection [19, 26]. However, the enzyme-linked immune assay (ELISA) has for a while, due to its ease, the routine diagnostic system for the dengue infection serological confirmation [27, 28]. Different kits are commercially available, such as Panbio Dengue Duo IgM and IgG Rapid Cassette test kits and commercial Platelia Dengue NS1 antigen capture ELISA kit. Clearly, the availability of systems for the detection of dengue infections is a public health priority. Therefore, in this study, we show the expression of a recombinant NS3 protein from all four serotypes of dengue virus and we report a reliable “in-house detection system” for the diagnosis of dengue infection that was field-tested in a small village (Tezonapa) in the state of Veracruz, Mexico. 

## 2. Materials and Methods

### 2.1. Cells and Viruses

*Aedes albopictus* cells (C6/36, ATCC:CRL-1660) were grown in Dulbecco's modified Eagle's medium supplemented with 10% heat-inactivated fetal bovine serum (DMEM-10% FBS), 0.29 mg/mL L-glutamine, 200 U/mL penicillin, and 0.2 mg/mL streptomycin. DENV-1 (Hawaii), DENV-2 (New Guinea C), DENV-3 (H87) and DENV-4 (H241) were obtained from the State Public Health Laboratory in Veracruz, Mexico. The virus stock was prepared by infecting C6/36 cell monolayer in 75 cm^2^ tissue culture flasks at 75%–85% confluence. After 2 h of DENV adsorption, 20 mL of minimum essential medium (MEM) supplemented with 10% FBS was added and the flasks were incubated at 28°C until the cytopathic effect was evident. Cells and supernatant were then harvested by gentle pipetting, clarified by centrifugation (1,200 ×g for 20 min), aliquoted, and stored at −80°C until required.

### 2.2. Preparation of NS3 Expression Constructs

 Viral RNAs were obtained by extracting 2 mL of the clarified culture media with 1 mL of TRIzol LS Reagent (Invitrogen) according to the manufacturer's instructions and used as template for the synthesis of a cDNA. The reaction was performed by reverse transcriptase SuperScript II (Invitrogen) with a primers DENV1–4 NS3 forward (DEN1, 5-GGGGGCGGAGGTAGTGGTGGAGGCGGGTCAGGAGTGCTATGGGACAC-3; DEN2,  5-GGGGGCGGAGGTAGTGGTGGAGGCGGGGCCGGAGTATTGTGGGATGT-3; DEN3, 5-GGGGGCGGAGGTAGTGGTGGAGGCGGGTCCGGCGTTTTATGGGACG-3; DEN4, 5-GGGGGCGGAGGTAGTGGTGGAGGCGGGTCAGGAGCCCTGTGGGAC-3) and DEN1–4 NS3 reverse (DEN1, 5-ATCGATGATCATTACCTAAACACCTCGTCCTCAATC-3;  DEN2, 5-TAATGGATCCTTACTTTCGAAAGATGTCATCTTCA-3; DEN3, 5-GGCGGATCCTTATGCATTTGTTTGCGCTATTCC-3; DEN4, 5-GGCGGATCCTTACTTTCGAAAAATGTCCTCATCC-3; restriction enzyme sites are underlined, DEN1 *Bcl*I, DEN2–4 *Bam*HI) [29]. The samples were subjected as follows: denaturation (94°C for 1 min), primer annealing (NS3-DEN1 at 58°C, NS3-DEN2 at 48°C, NS3-DEN3 at 60°C, and NS3-DEN4 at 55°C for 2 min), and primer extension (72°C for 2 min) followed by 30 cycles with an extension step of 7 min at 72°C. The different amplified products (500–600 bp) were electrophoresed on a 1.8% agarose gel, recovered with gel extraction Kit QIAEX II (QIAGEN, Germany). Four plasmids based on the pCR 2.1-TOPO vector (Invitrogen-Life Technologies) were constructed encoding the NS3pro185 sequence (domain protease). The recombinant vectors were restricted with *Eco*RI, and the fragments were ligated in frame into pGEX-5X-1 vector (Pharmacia) previously digested with this same enzyme. 

### 2.3. Expression, Solubilization, and Purification of GST-DVNS3-1, GST-DVNS3-2, GST-DVNS3-3, and GST-DVNS3-4

Competent *Escherichia coli* strain DH5-*α* cells were transformed with the parental vector (pGEX-5X-1) as with the recombinant expression vector (pGEX-DVNS3-1, 2, 3, or 4), were inoculated into LB media containing 100 mg/L ampicillin (Sigma, St. Louis, MO, USA), and incubated at 37°C overnight. Fresh LB media was incubated at 37°C with the overnight culture (1 : 100) to an OD_600_ = of 0.5, and protein production was induced by addition of isopropyl-*β*-D-thiogalactoside (IPTG) to a final concentration of 0.1 mM. After 2 h incubation, cells were harvested and purification of expressed proteins was performed essentially as described by López-Monteon et al. 2003 [30] with the following modifications. Then, pellets were treated to solubilize the inclusion bodies; briefly, the pellets were washed twice with 50 mL of PBS (137 mM NaCl, 2.7 mM KCl, 4.3 mM Na_2_HPO_4_·7H_2_O, and 1.4 mM KH_2_PO_4_, pH 7.4), incubated at 37°C under constant stirring for 20 min and centrifuged at 3,046 ×g at 4°C for 10 min. After that, pellets were suspended by vortexing with PBS 1X pH 7.4 containing 2 M urea, the sample was stirred vigorously for 2 min, incubated at 37°C under constant stirring for 30 min, and subsequently, centrifuged at 3,046 ×g for 10 min. The supernatants obtained from the solubilization of the inclusion bodies were dialyzed to remove urea. These supernatants were dialyzed against PBS 1X pH 7.4 overnight at 4°C with constant stirring. The supernatants containing solubilized fusion proteins (GST-NS3DEN1, 2, 3, and 4) were mixed with glutathione-agarose beads (sulfur linkage; Sigma). After adsorption for 30 min, beads were collected and washed by centrifugation. Either GST or GST-DVNS3-1, 2, 3, and 4 (rNS3) were eluted by competition with free glutathione (15 mM glutathione in 50 mM Tris-HCl pH 8.0) and then acetone-precipitated.

### 2.4. Immunization of Mice with GST-DVNS3-1, 2, 3, 4

 Female BALB/c mice (6- to 8-week old) were immunized by intraperitoneal route. All mice were maintained according to the recommendations by our Institutional Animal Care and Use Committee. The mice were immunized with one dose of 100 *μ*g of antigen and two more with 50 *μ*g. First immunizations were performed with the antigen emulsified in complete Freund's adjuvant (CFA), and re-immunizations at one-week intervals were performed with incomplete Freund's adjuvant (Gibco-BRL, Grand Island, NY, USA). The same schedule was used for control group, which received only GST plus adjuvant. At the end of the immunization scheme, animals were bled to obtain immune sera.

### 2.5. SDS-PAGE and Immunoblotting

 Proteins were resolved on 10% SDS-PAGE [31] and visualized by staining with Coomassie brilliant blue or electrophoretically transferred onto nitrocellulose paper for immunoblotting [32, 33]. Pooled sera from each group of immunized mice were used as primary antibodies at serial dilutions (1 : 100–1 : 3200) in TBS-T (150 mM NaCl, 0.05% Tween 20, 2% skim milk, and 10 mM Tris-HCl pH 7.4). Bound antibodies were detected using alkaline phosphatase-conjugated goat anti-mouse IgG (Pierce, Rockford, IL, USA) diluted at 1 : 5000, then developed with NBT and BCIP (Sigma).

### 2.6. Study Area and Sample Collection

 The study was conducted in the municipalities of Tezonapa from the sanitary jurisdiction of Cordoba, in central Veracruz, located at latitude 18°36′ north and longitude 96°41′ west. A total of 10 rural localities were included: Caxapa, El Mirador, El Suspiro, Las Josefinas, La Joya, La Luna, Paraíso La Reforma, Rancho Nuevo, Raya Caracol, and San Agustín del Palmar. These villages are at an altitude of 80–700 m and are located at the junction of the coastal plains of Veracruz on the east and mountains of the Trans-Mexican volcanic belt on the west. In each village, research personnel provided general information on dengue virus and the project to households during open meetings organized in the rural medical units through the IMSS-Opportunities social program. Interested participants were given an appointment for their family at the medical unit for blood draws. On the appointment day, a written informed consent was obtained from each volunteer, and research personnel collected the blood samples in vacutainers tubes. Serum was separated by centrifugation at 1,200 ×g for 10 min and samples were stored at −70°C until used.

### 2.7. Dengue Diagnostic

 A short questionnaire on knowledge of the disease and general clinical signs was also applied to the participants when blood samples were taken, with questions that included whether they have suffered fever higher than 37.5°C, headache, retro-orbital and abdominal pain, vomiting, skin rash, nose or any other type of hemorrhage. A total of 239 serum samples were collected and analyzed for dengue virus infection using four different tests, including Panbio Dengue IgM and IgG Capture ELISA (Panbio Diagnostics, Brisbane, Australia), Platelia Dengue NS1 antigen capture ELISA (Bio-Rad) detection and in-house system (anti-rNS3), and RT-PCR assay specific for a fragment of the protein E from dengue virus.

### 2.8. Dengue Diagnosis by an In-House System

 To test all serum samples, an indirect ELISA method was carried out as follows: 96-well plates were coated with 100 *μ*L of carbonate buffer (pH 9.6) containing the antigen at concentration 2 *μ*g/mL (pool of four recombinant NS3 proteins rNS3). After overnight 4°C incubation, the plates were washed five times with PBS-T (0.05% Tween-20 in phosphate buffer solution), five minutes per wash, and blocked with 5% skimmed milk in PBS for 45 min at room temperature. Serum samples were serially diluted; the dilution that generated an OD value three times higher than that from negative samples was thoroughly used in the rest of the study. All serum samples were diluted 1 : 50, and 100 *μ*L of each one was added to individual wells in triplicate and incubated for two hours at 37°C. Further washing steps were conducted, and a peroxidase-labeled goat anti-human IgG antibody (Pierce, Rockford, IL, USA) was added at a 1 : 8,000 dilution in PBS/0.05% Tween 20 and incubated for 1 hour at room temperature. After eight washes, 100 *μ*L of 2,2,-azino-bis(3-ethylbenzthiazoline)-6-sulphonic acid (Zymed, South San Francisco, CA, USA) was added as substrate and the reaction was allowed to proceed for 20 min at room temperature. The reaction was stopped with 2% sulfuric acid, and absorbance was read at 415 nm with an ELISA microplate reader (Multiscan MS; Labsystems, Vantaa, Finland). The cutoff (0.21) for this assay (at dilution 1 : 100) was established using the average obtained from a sample of 25 apparently healthy human sera plus two standard deviations (SDs). Positive samples were defined as samples with absorbance greater than two SDs above the mean of the negative control.

### 2.9. Viral RNA Extraction

 To study the molecular typing of DENV, attempts were made to isolate the RNA from all the sera samples as well as from four different DENV serotype strains were performed, which were used as positive control. Using TRIzol-SL according to the manufacturer's protocol isolated viral RNA.

### 2.10. RT-PCR Serotyping Dengue Virus

 In a single tube, viral RNA was converted to a DNA copy (cDNA) prior to enzymatic DNA amplification by the use of reverse transcriptase (RT) and the DENV downstream consensus primer D2-5′-TTGCACCAACAGTCAATGTCTTCAGGTTC-3′ homologous to the genomic RNA of the four serotypes. cDNA was synthesized using SuperScript II kit (Invitrogen) in a 20 *μ*L reaction mixture containing 4 mM dNTPs (Applied Biosystems), 4 mM MgCl_2_ (Applied Biosystems), 1 U/*μ*L RNase inhibitor, 2.5 U/*μ*L reverse transcriptase (Invitrogen), and 1 *μ*g RNA. The reaction mixture was incubated for 30 min at 42°C and then the transcriptase was inactivated at 70°C for 5 min. 5 *μ*L from obtained cDNA was used as template in a 25 *μ*L reaction mixture containing 1.5 mM MgCl_2_, 2.5 mM dNTPs, 1X PCR Buffer II, and 2.5 U of AmpliTaq Gold DNA polymerase with 20 pmol dengue virus group-specific consensus primers (D1: 5′-TCAATATGCTAAAACGCGCGAGAAACCG-3′ and D2: 5′-TTGCACCAACAGTCAATGTCTTCAGGTTC-3′). The PCR (511 bp) was carried out under the following conditions: 28 cycles of 94°C for 30 s, 55°C for 1 min, and 72°C for 2 min, and then an extension step at 72°C for 7 min. 

DENV serotyping was conducted by second-round amplification (nested PCR) initiated with 1 *μ*L of diluted material (1 : 40 in sterile distilled water) from the initial amplification reaction. The total 25 *μ*L of reaction mixture was prepared using 1 *μ*L of diluted first PCR products, 1.5 mM MgCl_2_, 2.5 mM dNTPs, 0.5 U of Taq DNA polymerase, and 20 pmol of primer D1 and 20 pmol of dengue virus type-specific primers (Ts1: 5′-CGTCTCAGTGATCCGGGGG-3′, Ts2: 5′-CGCCACAAGGGCCATGAACAG-3′, Ts3: 5′-TAACATCATCATGAGACAGAGC-3′, and Ts4: 5′-TGTTGTCTTAAACAAGAGAGGTC-3′), as reported earlier [34]. The samples were subjected to initial denaturation (95°C for 3 min) followed by 20 cycles of denaturation (95°C for 30 s), primer annealing (55°C for 1 min), and primer extension (72°C for 2 min) along with final extension (72°C for 7 min). The PCR products were analyzed by running a 1.8% agarose gel stained with ethidium bromide. The sizes of fragments were DENV-1 (482 bp), DEN-2 (119 bp), DENV-3 (290 bp), and DENV-4 (392 bp). 

### 2.11. Data Analysis

 All proportion data are presented with a 95% confidence intervals (CIs). Using Fisher's exact tests we compared proportion data, and the kappa index was calculated when applicable. The relationship between age and seroprevalence rate was assessed by chi-square test and by regression analysis. All villages were georeferenced and a spatial database of serologic results was created in ArcView 3.2 (Environmental Systems Research Institute, Redlands, CA, USA) to produce seroprevalence maps.

## 3. Results

### 3.1. Expression, Purification and Immunogenicity of Recombinant GST-DVNS3-1, GST-DVNS3-2, GST-DVNS3-3, and GST-DVNS3-4 Proteins

**Figure 1:**
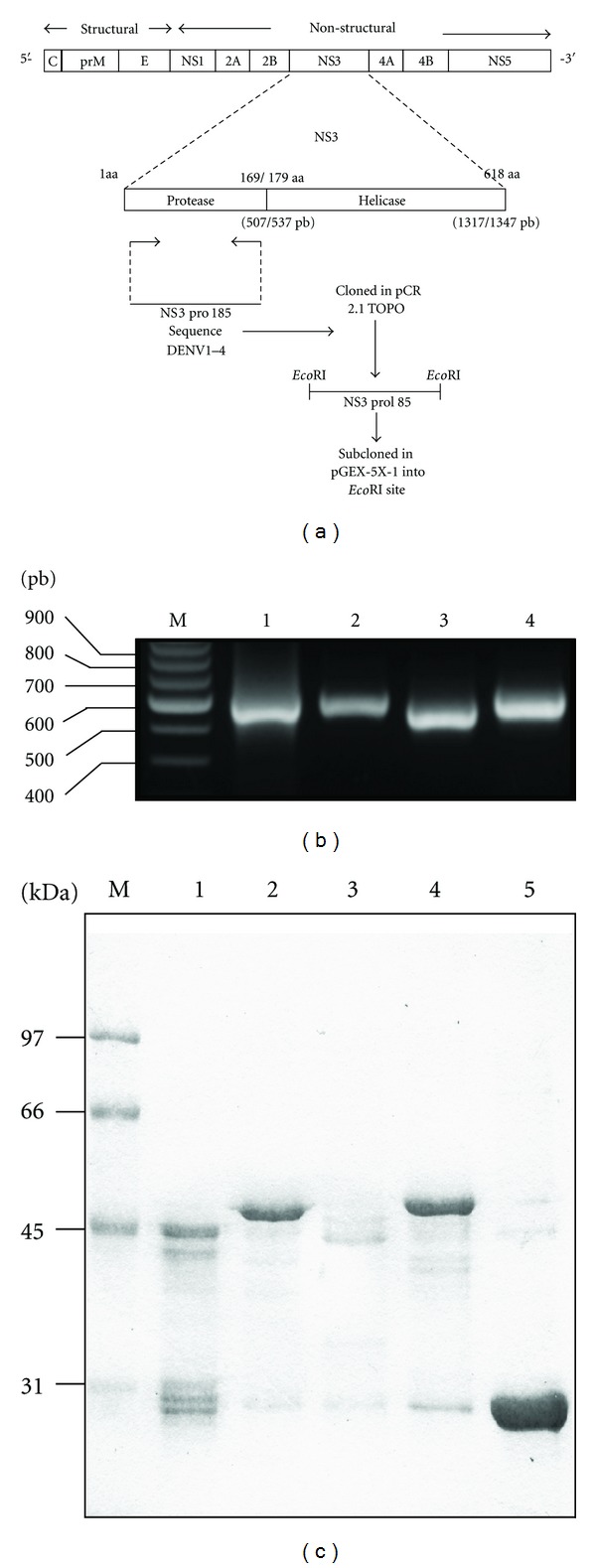
Obtaining recombinant NS3 protein. (a) Cloning strategy for the NS3 domain protease of dengue virus in the cloning vector and in prokaryotic expression vector. (b) Amplification of the NS3 protease gene cloned into pCR2.1TOPO, (M) size marker, Lane 1, NS3-DEN1. Lane 2, NS3-DEN2. Lane 3, NS3-DEN3. Lane 4, NS3-DEN4. (c) Purification of the recombinant protein GSTNS3 of each serotype. (M) molecular weight marker. Lane 1, GST-NS3DEN1. Lane 2, GST-NS3DEN2. Lane 3, GST-NS3DEN3. Lane 4, GST-NS3DEN4. Lane 5, GST.

Four DNA fragments were obtained from the cDNA encoding for NS3 domain protease (NS3pro185 sequence) from all serotypes of DENVs by PCR (Figure 1(a)). All fragments (NS3-DEN1/597 bp, NS3-DEN2/596 bp, NS3-DEN3/590 bp, NS3-DEN4/598 bp) (Figure 1(b)) were cloned into pCR2.1TOPO vector and subcloned again in correct open reading frame with orientation into pGEX-5X-1 expression vector (Figure 1(a)). The expression of the recombinant proteins pGEX-NS3DEN1, pGEX-NS3DEN2, pGEX-NS3DEN-3, pGEX-NS3DEN4, and pGEX-5X-1 (parenteral plasmid) was evaluated in *E. coli* DH5*α* cells upon induction with IPTG at 37°C. The proteins were purified from inclusion bodies. The amount of GST-fusion proteins present in the fractions corresponded to a yield between 1.8 and 5 mg/L culture depending on the recombinant protein; GST-NS3DEN3 protein expression was the lowest (1.8 mg/L).  Figure 1(c) shows the fusion proteins obtained after 2 hours of induction and after the inclusion bodies were purified by solubilization with 2 M urea. After purification by affinity chromatography and analysis by SDS-PAGE, results revealed the expression of a novel protein of ~49 kDa (Figure 1(c), lane 1–4) and expression of GST as a 27-kDa protein (Figure 1(c), lane 5). They were detected by western blotting using a polyclonal antibody against GST proteins, and all of them had the predicted weight. 

In order to establish that these purified GST dengue proteins can induce humoral immune response, groups of 5 BALB/c mice were immunized with recombinant proteins. Serum samples were analyzed at the end of the immunization scheme. The fusion proteins were immunogenic, inducing antibodies able to recognize antigens up to 1 : 3200 dilution (data not shown).

### 3.2. Serology and RT-PCR DENV Serotyping

**Figure 2:**
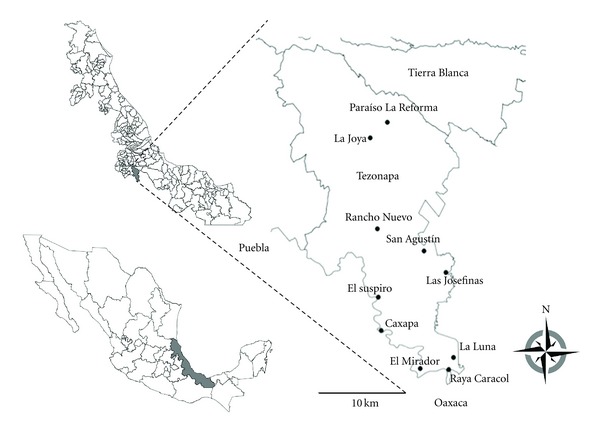
Study area. Mexico (bottom left), the state of Veracruz (center), and the study area (inset), corresponding to municipality of Tezonapa. Black circles show the position of the indicated villages.

**Figure 3:**
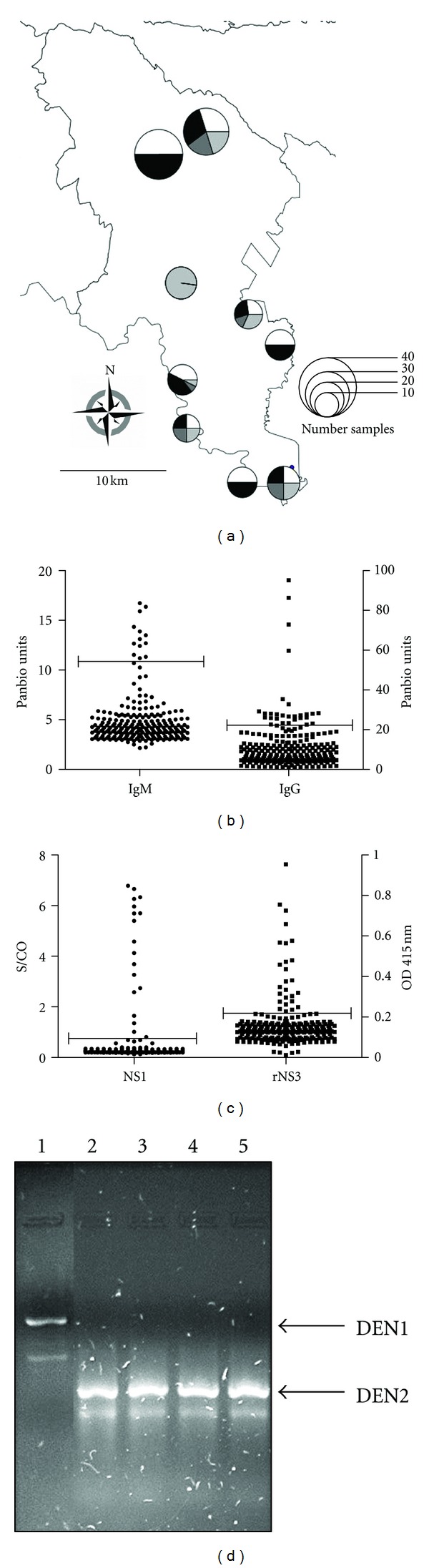
Seroprevalence and serotyping of dengue virus. (a) Geographical distribution of cases in the municipality of Tezonapa; the circles on the map indicate the villages, with their size proportional to the number of samples as indicated on the map, white areas represent IgG-positive samples, dark gray areas represent IgM-positive samples, light gray areas represent NS1-positive samples, and black areas represent NS3-positive samples. (b) Identification of positive samples for IgM and IgG by ELISA (Panbio). (c) NS1 antigen detection (Bio-Rad) and identification of NS3-positive samples by ELISA (in-house detection system). (d) Serotyping of dengue virus by RT-PCR, lanes 1–5 representative samples from rural villages; heat arrow indicates PCR product by DEN 1 and DEN 2.

 A total of 239 serum samples were collected from an area in Tezonapa, Veracruz (Figure 2), mostly were woman (*n* = 166, 69.4%). Age of participants ranged from 3 to 65 years. All 239 samples were tested for dengue IgM and IgG antibodies, NS1 antigen detection, and anti-NS3 antibodies (Figure 3(a)). A total of 13 samples were positive for IgM, obtaining a seroprevalence of 5.43% (95% CI = 4.23–6.63%). When analyzing the presence of IgG, a total of 28 samples were positive, obtaining a seroprevalence of 11.7% (95% CI = 10.26–13.16%) (Figure 3(b)). Seventeen samples were positive for NS1 capture was observed 7.11% (95% CI = 5.59–8.73%) (Figure 3(c)). When analyzing the prevalence of anti-DENV antibodies using a pool of recombinant NS3 proteins (rNS3), a total of 26 samples were positive and seroprevalence was observed of 10.87% (95% CI = 9.42–12.32%) (Figure 3(c)). The same samples that were positive to NS1 antigen proved positive PCR for DENV (1 sample for serotype 1 corresponding to Paraíso la Reforma and 16 samples for serotype 2 to San Agustín del Palmar) observing an overall infection rate of 7.11% (95% CI = 5.59–8.73%) (Figure 3(d)). 

### 3.3. In-House Dengue System Evaluation

 Eight samples were positive for the five tests; 7 samples were positive for four tests. The concordance between the three ELISA evaluations was very good. IgM Panbio ELISA versus rNS3 ELISA, *κ* = 0.934 ± 0.064 (95% CI = 0.808−1.061). The value of protein as a possible diagnostic test was evaluated, the observed agreement IgG Panbio ELISA versus rNS3 ELISA, *κ* = 0.872 ± 0.048 (95% CI = 0.779–0.965), was very good (observing an equal number of positive samples in both tests; *P* ≤ 0.0001, by Fisher's exact test), a sensitivity of 0.9231 (95% CI = 0.7488–0.9905), a specificity of 0.9812 (95% CI = 0.9526–0.9949), a positive predictive value of 0.8571 (95% CI = 0.6730–0.9597), and negative predictive value of 0.9905 (95% CI = 0.9661–0.9989). Prevalence rate was not significantly correlated with age (*r* ^2^ = 0.8024; *P* = 0.3761, by second-order polynomial regression) (data not shown). The agreement between the NS1 antigen detection versus rNS3 ELISA, *κ* = 0.837 ± 0.066 (95% CI 0.708–0.966), was very good, a sensitivity of 1.000 (95% CI = 0.7708–0.9946), a specificity of 0.9595 (95% CI = 0.9219–0.9801), a positive predictive value of 0.6538 (95% CI = 0.4436–0.8206), and negative predictive value of 1.000 (95% CI = 0.9779–0.9996).

## 4. Discussion

Dengue infection is a growing public health concern in endemic areas all over the world. Hyperendemic geographical areas have been defined as those with continuous presence of multiple viral serotypes and competent vectors, and a large population of susceptible hosts, as it seems to be the case for Mexico [1]. Despite the importance of the DENVs as emerging pathogens, diagnostic tests remain inadequate for efficient and accurate identification of DENV infection. Mexico, currently, has a network of public health laboratories which consists of 30 laboratories that perform confirmation diagnosis of dengue by using immunoassay techniques for detection of viral antigen NS1, IgM antibody, or IgG, depending on the time evolution of the disease when the patient seeks medical care. It is well recognized that there is a high level of underreporting of cases of dengue even in areas where there is an adequate surveillance system. It is expected that the level of underreporting in dispersed rural areas and with poor access to health services is even higher [35]. Our study focused on diagnosis of dengue virus infection as well as on the presence of antibodies in rural areas of central Veracruz state.

Current dengue diagnostic methods have a number of serious limitations. The gold standard for diagnosis of acute DENV infection is viral isolation, but the procedure is costly (US$39.10), time consuming, and technically difficult to perform. Reverse transcriptase PCR (RT-PCR) has been widely adopted as an alternative for viral isolation in the diagnosis of acute infection, but PCR is technically intensive and expensive (US$136.67), and its sensitivity varies from 80 to 90% based on primer sets. An IgM antibody capture enzyme-linked immunosorbent assay (MAC-ELISA) is useful primarily for diagnosing dengue infection in the late acute or early convalescent phase of the illness but is often insensitive for early-acute-phase infections [36]. The distinction between primary and secondary infections is currently assessed by measuring IgM and IgG responses to dengue antigens in paired serum samples taken from a febrile patient in the acute stage of disease and after convalescence [37]. In order to set up a rapid and reliable diagnosis, some laboratories in Mexico use the Panbio Dengue Duo IgM and IgG Rapid Cassette test kits or the ELISA Dengue IgM capture kits. However, it is sometimes difficult to have access to these kits due to stock shortage in the market, problems to introduce foreign products, and of course, high cost. Therefore it is necessary to find alternatives for the diagnosis of DENV during the acute phase of disease, because diagnostic tests remain inadequate for efficient and accurate identification of DENV infection. The objective of this work was to study the expression of a recombinant NS3 protein from all four serotypes of dengue virus and report a reliable “in-house detection system” for the diagnosis of dengue infection and compared to commercially available kits (Panbio Dengue Duo IgM and IgG Rapid Cassette test kits and commercial Platelia Dengue NS1 antigen capture ELISA kit). The use of recombinant NS3 protein as a diagnostic method for identifying primary and secondary infections provided very good results, obtaining a good concordance between the tests for both IgM and IgG (Panbio IgM/IgG test versus rNS3 ELISA), and when we perform the study of the predictive ability of a diagnostic test, it was observed a good specificity and sensitivity with ELISA rNS3. Similarly, very good agreement is observed when comparing the NS1 antigen detection versus rNS3 ELISA. Nonstructural protein 1 (NS1) of DENV has been shown previously to be useful as a tool for the diagnosis of acute dengue infections. NS1 has been detected in the sera of DENV-infected patients as early as the first day of the symptoms and up to 18 days [38]. Our results showed that all samples that were positive for antigen detection NS1 were also positive for the presence of antibodies against the recombinant NS3 protein. These results suggest that the use of ELISA with recombinant NS3 protein may be an alternative method for serological analysis of dengue virus in the acute phase. NS3 protein plays a predominant role in the pathogenesis of the disease and together with its role during the processing of the precursor of the viral polyprotein makes it an interesting protein for evaluating host responses. Some authors have reported a significant antibody response to NS3 in both primary and secondary infections [39–41]. However, Valdes et al., 2000 [40], showed a significant antibody response in secondary cases when the infecting serotype was DEN2. In this case, in-house detection system used an anti-human IgG antibody as second antibody, which allowed to suggest that antibodies present in the sera of patients were IgG but not IgM antibodies. This is consistent with the findings from Valdes et al. [40], as we found that IgG antibodies for a secondary response and virus serotyping results show that the major infecting serotype was DEN2. The conclusion that the use of ELISA with recombinant NS3 protein may be an alternative method for serological analysis of dengue virus in the acute phase results from the fact that there is an excellent concordance when comparing the NS1 antigen detection versus rNS3 ELISA.

It has been reported that dengue is transmitted in rural areas and currently has become condition of urban nature. In addition, migrations of population favor a permanent risk for the spread of dengue-endemic areas. 

The characteristics of an “ideal” dengue diagnostic test depend on the purpose for which the test will be used; in case of early diagnosis the dengue test should distinguish between dengue and other diseases with similar clinical aspects (such as malaria, leptospirosis, and typhoid), highly sensitive during the acute stage of infection, provides rapid results, inexpensive, easy to use, and stable at temperatures greater than 30°C for use in rural areas, if necessary [42]. Additionally to this, the study of the predictive ability of a diagnostic test on the in-house dengue system was observed with a good specificity and sensitivity and provides rapid results (3 hours); moreover it is less expensive than commercial tests available in the market. Serological tests have an intermediate price compared to molecular tests. In fact, when quoting a new test, the laboratories indicated that it should be considered a 4–7-fold increase versus the reagent price. In this sense, the cost assessment of “in-house” ELISA and commercial ELISA kits showed that “in-house” ELISA proved to be a cheaper kit than the commercial ones, with a cost of US$8.20, while other kits have higher costs as it is showed next: NS1 antigen detection (US$18.80), IgM (US$22.62), and IgG capture ELISA (US$24.04). This share reflects the actual costs of each test method based on the actual cost of reagents, laboratory supplies, stationery, personnel, and indirect costs as electricity, water, depreciation of equipment, and so forth.

Finally, the identification of the circulation of more than one serotype in the municipality of Tezonapa (DEN1 and DEN2) is a warning sign because it may favor the increase of severe clinical forms of dengue due to the presence of secondary infection by different serotypes, promoted by the phenomenon of increased antibody-dependent infection. We propose the use of recombinant NS3 protein as a detection system as a viable alternative in Mexico, when commercial kits are not available, at least in terms of a first screening. Even if this system needs to be improved by increasing the number of samples, for example, specially from asymptomatic individuals from the endemic regions in Mexico, to increase its accuracy in the dengue virus diagnosis, it is important to keep working on the development of reliable diagnostic tools in order to establish an efficient surveillance system in dengue endemic areas.
